# Local clustering via approximate heat kernel PageRank with subgraph sampling

**DOI:** 10.1038/s41598-021-95250-w

**Published:** 2021-08-04

**Authors:** Zhenqi Lu, Johan Wahlström, Arye Nehorai

**Affiliations:** 1 Preston M. Green Department of Electrical and Systems Engineering, Washington University in St. Louis, St. Louis, MO USA; 2 Department of Computer Science, University of Exeter, Exeter, UK

**Keywords:** Physics, Statistical physics, thermodynamics and nonlinear dynamics, Complex networks

## Abstract

Graph clustering, a fundamental technique in network science for understanding structures in complex systems, presents inherent problems. Though studied extensively in the literature, graph clustering in large systems remains particularly challenging because massive graphs incur a prohibitively large computational load. The heat kernel PageRank provides a quantitative ranking of nodes, and a local cluster can be efficiently found by performing a sweep over the heat kernel PageRank vector. But computing an exact heat kernel PageRank vector may be expensive, and approximate algorithms are often used instead. Most approximate algorithms compute the heat kernel PageRank vector on the whole graph, and thus are dependent on global structures. In this paper, we present an algorithm for approximating the heat kernel PageRank on a local subgraph. Moreover, we show that the number of computations required by the proposed algorithm is sublinear in terms of the expected size of the local cluster of interest, and that it provides a good approximation of the heat kernel PageRank, with approximation errors bounded by a probabilistic guarantee. Numerical experiments verify that the local clustering algorithm using our approximate heat kernel PageRank achieves state-of-the-art performance.

## Introduction

Networks are a standard representation of complex interactions among multiple objects, and network analysis has become a crucial method for understanding the features of a variety of complex systems^1–8^. *Graph clustering* is a fundamental technique for understanding mesoscopic structures in networks consisting of *communities* or *clusters*, that is, groups of nodes that are densely connected locally but sparsely connected to other groups in the network^9^. The problem of graph clustering has been studied extensively in the literature, and the solutions to this problem have found many applications^10–15^. Yet, as real-world networks become larger and larger, clustering massive graphs incurs a huge computational load, since the numbers of computations of many existing clustering algorithms are at least linearly dependent on the size of graphs. At the same time, in a variety of situations, we may be interested only in one single cluster near one object in the network. For these situations, we can instead make use of local algorithms to perform local clustering.

The goal of local clustering is to identify, near a specified seed node, a subgraph that is more densely connected internally than to the rest of the graph. A local clustering algorithm utilizes only the local structure: it starts from the seed node, and examines only nodes connected by an edge to those it has examined before. The first local clustering algorithm was proposed by Spielman and Teng^16,17^, and employed random walks on graphs to explore the local structure near the seed node. The idea behind this algorithm is that nodes near the seed node are more likely to be visited by random walks starting from the seed node. Thus such likelihoods probe the local structure near the seed node, and a local cluster can be found by simulating random walks on graphs and then evaluating resulting likelihoods.

Andersen et al. improved the algorithm of Spielman and Teng by examining approximate PageRank vectors^18–20^. A PageRank vector is a geometric sum of the probability distributions induced by random walks starting from a specified initial distribution. If the initial distribution is concentrated on the seed node, then the PageRank vector provides a ranking of nodes at which random walks starting from the seed node most likely end. The set of nodes with high rankings approximates a good local cluster near the seed node.

State-of-the-art local clustering algorithms are based on *heat kernel PageRank*^21^. Similar to standard PageRank, heat kernel PageRank is based on random walks. However, heat kernel PageRank has the special benefit of also satisfying the heat equation^22^. The exponential coefficients of heat kernel PageRank decay much faster than the geometric coefficients of standard PageRank, and so shorter random walks are more heavily weighted in heat kernel PageRank^23^. Subsets obtained by using heat kernel PageRanks are guaranteed to have good conductance values^22^, which are commonly used as goodness measures for clusters and communities. As with standard PageRank, computing an exact heat kernel PageRank vector is prohibitively computationally expensive, and most methods use approximate algorithms instead^21,24^. Yet these algorithms still compute the heat kernel PageRank vector on the whole graph, and thus the number of computations is dependent on the size of the whole graph.

In this paper, we present a local algorithm for approximating the heat kernel PageRank. Instead of working on the whole graph, we compute the values of the heat kernel PageRank vector only for nodes in a subgraph near the seed node. This subgraph is obtained by sampling nodes near the seed node in a greedy way. We then simulate random walks inside this sampled subgraph and evaluate the probability distributions resulting from these random walks. We show that, by making a few mild assumptions about the graph, the approximation errors induced by restricting the random walks in a subgraph can be well bounded and do not cause significant changes to the heat kernel PageRank vector. Finally, we apply the proposed local clustering algorithm to computer-generated benchmark networks and real-world networks. When applied to networks with known communities, our local clustering algorithm finds local clusters that show excellent agreement with the ground-truth communities. When applied to real-world networks without a-priori information of communities, our local clustering algorithm yields promising results that aid our understanding of the organization and structure in these massive complex networks.

## Preliminaries

Let $G=(V,E)$ be an undirected simple graph on *n* nodes, where *V* is the set of nodes and *E* is the set of edges. We use $u\sim v$ to denote that a pair of nodes *u* and *v* are connected by an edge $\lbrace u,v \rbrace \in E$, and the nodes in such a pair are called neighbors. The *degree* $d_v$ of a node *v* is the number of edges attached to the node *v*. The *volume* of a subgraph $S\subset V$ is defined as the sum of the degrees of its nodes,

1 $$\begin{aligned} {\mathrm{vol}}(S) = \sum _{v\in S}d_v, \end{aligned}$$

which is also known as the total degree of subgraph *S*. The *edge boundary* $\partial (S)$ of subgraph *S* is the set of edges with one end in *S* and another end not in *S*,

2 $$\begin{aligned} \partial (S) = \lbrace u\sim v : u\in S, v\notin S \rbrace . \end{aligned}$$

For a given subgraph *S* of a graph *G*, the *conductance* value $\Phi (S)$, also called the *Cheeger ratio*, of the induced cut $(S,V{\setminus } S)$ is defined as

3 $$\begin{aligned} \Phi (S) := \frac{|\partial (S)|}{\min ({\mathrm{vol}}(S),{\mathrm{vol}}(V{\setminus } S))}, \end{aligned}$$

which is the ratio of the number of edges between the two parts of the cut and the volume of the smaller part of the cut. This ratio can also be interpreted as the fraction of edges leaving the small part of the cut. We will use this ratio as the objective function to search for a local cluster near a seed node.

Let $p:V \rightarrow [0, 1 ]$ be a probability distribution vector over the nodes. Consider a probability-per-degree ranking of the nodes in the graph where

4 $$\begin{aligned} \frac{p(v_1)}{d_{v_1}} \ge \frac{p(v_2)}{d_{v_2}} \ge \cdots . \end{aligned}$$

Let $S_i$ be the set of the first *i* nodes per the ranking:

5 $$\begin{aligned} S_i = \lbrace v_1,v_2,\dots ,v_i \rbrace . \end{aligned}$$

The set $S_i$ is called a *segment*. The process of investigating all local subgraphs induced by segments to find an optimal local community is called *performing a sweep* over the probability distribution vector *p*. The $\varsigma$-local Cheeger ratio $\Phi _{\varsigma }(p)$ of a sweep over a vector *p* is the minimum Cheeger ratio among all segments with volume from 0 to $2\varsigma$. For any subgraph *S*, we define *p*(*S*) to be the sum of all values of *p*(*v*) for $v \in S$:

6 $$\begin{aligned} p(S) := \sum _{v \in S} p(v). \end{aligned}$$

A *heat kernel PageRank* vector $\rho _{t,f}$ is defined as

7 $$\begin{aligned} \rho _{t,f} := \sum _{k=0}^{\infty }e^{-t}\frac{t^k}{k!}f\,P^k, \end{aligned}$$

where *t* is a non-negative real value representing temperature, $f:V\rightarrow {\mathbb {R}}$ is a preference vector on the graph representing the starting state of the heat diffusion, and *P* is the transition probability matrix

8 $$\begin{aligned}{}[P ]_{uv} = \left\{ \begin{array}{ll} 1/d_u &{}\quad {\text {if}}\; u\sim v,\\ 0 &{}\quad {\text {otherwise}}. \end{array} \right. \end{aligned}$$

If *f* is a probability distribution vector, properly normalized so that $\sum _{v\in V}f(v)=1$, then the heat kernel PageRank vector $\rho _{t,f}$ can be regarded as the expectation of the ending state of a random walk whose length follows a Poisson distribution with mean *t* and whose starting state is *f*. This observation sheds light on an approximate calculation of the heat kernel PageRank vector: we simulate sufficiently many random walks with lengths following a Poisson distribution with mean *t* and record the nodes at which these random walks end.

## Sampling local subgraphs

Most local clustering algorithms calculate an approximate heat kernel PageRank vector on a whole graph, and thus the number of computations is dependent on the size of the whole graph. For example, the state-of-the-art method by Chung and Simpson requires ${\mathrm{O}}(\log n)$ computations^21^, where *n* is the size of the whole graph. We improve on the work of Chung and Simpson by stipulating that all simulated random walks must be local, i.e., we simulate random walks only around the seed node, to avoid unnecessary computations irrelevant to the local clustering that we wish to perform.

In the local clustering problem, given a seed node *s*, we wish to find a local cluster with a low conductance value around *s*. Based on this problem formulation, we propose to perform a rough sampling in the neighborhood of the seed node *s* and find a local subgraph. The local cluster of interest will be a subset of the sampled subgraph, and will occupy a non-vanishing fraction of this subgraph. Then we calculate an approximate heat kernel PageRank vector by simulating random walks in the sampled subgraph. Through this process, we can ensure that the number of computations of the heat kernel PageRank approximation is dependent only on the size of the local cluster, and not on the size of the whole graph.

Our proposed subgraph sampling algorithm is formulated as follows. We start with the seed node *s* and all its neighbors. Then we iteratively add nodes that roughly cause the greatest decrease, or the least increase, in the conductance value into a subgraph *S*, thereby expanding *S* in a greedy way while keeping the conductance value $\Phi (S)$ low. In each iteration, we investigate all neighboring nodes of the current subgraph *S*, namely all nodes with connections to nodes in *S*. We compute for each neighboring node *v* the fraction of connections to *S* in its total connections $d_v$. Then we add the nodes with the largest fraction into the subgraph *S*. We repeat this process iteratively until the size of the subgraph reaches the expected size, which is chosen to be proportional to the expected size $\varsigma$ of the local cluster of interest. Algorithm 1 provides the details of our subgraph sampling algorithm.
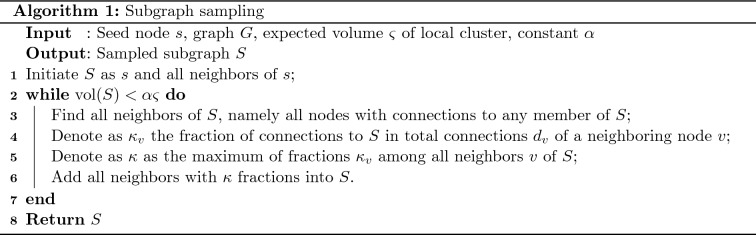


## Approximate heat kernel PageRank in sampled subgraph

We then calculate an approximate heat kernel PageRank vector on the support of the sampled local subgraph *S*. As stated earlier, we perform this calculation by simulating random walks only in subgraph *S*. Recall that the heat kernel PageRank vector $\rho _{t,s}$ on graph *G* with the starting preference vector $\chi _s$ is given as

9 $$\begin{aligned} \rho _{t,s} = \sum _{k=0}^{\infty }e^{-t}\frac{t^k}{k!}\chi _sP^k, \end{aligned}$$

where

10 $$\begin{aligned} \chi _s(u) = \left\{ \begin{array}{ll} 1 &{}\quad {\text {if}}\; u = s,\\ 0 &{}\quad {\text {otherwise}}, \end{array} \right. \end{aligned}$$

and *s* is the seed node. Since $\chi _s$ is properly normalized on graph *G*, the heat kernel PageRank vector $\rho _{t,s}$ is thus the expectation of the ending state of a random walk starting from the seed node *s* and tracing a length that follows a Poisson distribution with mean *t*. Based on this observation, the intuition of our algorithm of approximating $\rho _{t,s}$ on subgraph *S* works as follows. We perform *r* random walk simulations to approximate the infinite sum in (9) on the support of the sampled subgraph *S*, and choose *r* large enough to bound the error. In each simulation, we track the steps of the simulated random walk. If this random walk leaves the subgraph *S*, we terminate this simulation and restart. We also note that the Poisson distribution is highly concentrated around the mean value *t*, and thus we can choose a finite number *K* and discard all random walks with length greater than *K*.

Our proposed algorithm for the approximate heat kernel PageRank computation is summarized in Algorithm 2. Starting from the seed node, we generate *r* random walks, with lengths following a Poisson distribution. When a random walk reaches a node *v* in the subgraph *S*, one of the two following cases can happen. With probability proportional to the number of edges connecting to the outside of *S*, this random walk is terminated. Also, with probability proportional to the number of edges connecting to other nodes in *S*, this random walk chooses one of the neighbors of *v* in *S* at random as the next step. If a random walk is not terminated in the middle of the simulation, the ending node is recorded in the PageRank vector. The choice of the number of iterations *r* depends on an error tolerance parameter $\epsilon$, as we will discuss in detail later.
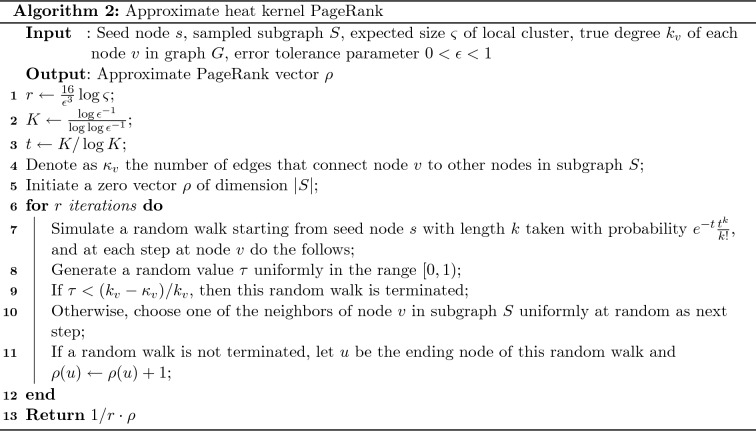


Restricting our attention to subgraph *S* comes with a price: we lose track of simulated random walks once they cross the boundary of *S* and enter the complementary part $V{\setminus } S$. But we are interested only in the part of the heat kernel PageRank vector that is supported on the subgraph *S*, and we can simply discard the other part that is supported on $V{\setminus } S$, since it is irrelevant to the local clustering we wish to perform. Thus, the only random walks that will interrupt our approximation of the heat kernel PageRank are those that leave the subgraph *S* first and then come back. But we will argue that, with reasonable assumptions, the contribution of these random walks is vanishingly small if the whole network is sufficiently large.

We will consider a network model with community structure as follows. The network is divided into a few non-overlapping communities, and each node belongs to one of these communities. Each node *v* has a mixing parameter $\mu _v \in (0, 1)$, such that a fraction $1 - \mu _v$ of its edges connects to other nodes in its community, and a fraction $\mu _v$ of its edges connects to other nodes outside its community. We additionally assume that $\mu _v$ is an independent and identically distributed (i.i.d.) random variable, with an expected value $\mu$. We note that our assumptions fit with commonly used network models with community structure, for example, the Girvan-Newman benchmark^25^, the LFR benchmark^26^, and the stochastic blockmodel^27^.

For a fixed seed node *s* in a local cluster of interest *C*, and for any node *v* in this community, we denote by $X_k^v$ the indicator random variable defined as $X_k^v=1$ if a random walk with length *k* starting from the seed node *s* ends at node *v*. By definition, the expectation ${\mathrm{E}}[X_k^v ]$ is equal to the fraction of random walks with length *k* starting from the seed node *s* that ends at node *v*. We denote by $X^v$ the random variable corresponding to the combined random walk process that starts from the seed node *s*, ends at node *v*, has a length that follows a Poisson distribution with mean value *t* and a maximum value of *K*, and has all steps in this cluster *C*. Then we can write $X^v$ as

11 $$\begin{aligned} X^v := \sum _{k\le K}e^{-t}\frac{t^k}{k!}X_k^v(1-\mu _{t_1})(1-\mu _{t_2})\cdots (1-\mu _{t_k}), \end{aligned}$$

where $t_1,\dots ,t_k$ are the nodes visited in a random walk of length *k*. This random variable $X^v$ is a weighted sum of the random variables $X_k^v$. The values of the weights follow a Poisson distribution corresponding to the distribution of the lengths of random walks, and are multiplied by a sequence of factors of the form $1-\mu$, which restricts all the steps of random walks to within subgraph *S*. The expected value of $X^v$ is

12 $$\begin{aligned} {\mathrm{E}}[X^v ]= \sum _{k\le K}e^{-t}\frac{t^k}{k!}(1-\mu )^k {\mathrm{E}}[X_k^v ]. \end{aligned}$$

This expected value represents the fraction of random walks starting from the seed node *s* that end at node *v*, which have lengths distributed according to a Poisson distribution with mean value *t* and a maximum value of *K*, and have all steps in this cluster *C*.

We will also consider the approximate form of the infinite sum in (9) by limiting the length of random walks to at most *K*. Let $\rho (K)_{t,s}$ denote the contribution to $\rho _{t,s}$ from random walks with a length of at most *K*,

13 $$\begin{aligned} \rho (K)_{t,s} = \sum _{k\le K}e^{-t}\frac{t^k}{k!}\chi _sP^k. \end{aligned}$$

It follows from definition that

14 $$\begin{aligned} \rho (K)_{t,s}(v) = {\mathrm{E}}\left[\sum _{k\le K}e^{-t}\frac{t^k}{k!}X_k^v \right]= \sum _{k\le K}e^{-t}\frac{t^k}{k!}{\mathrm{E}}[X_k^v ], \end{aligned}$$

which is the expected value of the random variable $Y^v$ corresponding to the combined random walk process that starts from the seed node *s*, ends at node *v*, and has length distributed according to a Poisson distribution with mean value *t* and has a maximum value of *K*:

15 $$\begin{aligned} Y^v := \sum _{k\le K}e^{-t}\frac{t^k}{k!}X_k^v. \end{aligned}$$

It follows that $\rho (K)_{t,s} = {\mathrm{E}}[Y^v ]$. So the value $\rho (K)_{t,s}(v)$ represents the fraction of random walks starting from the seed node *s* that end at node *v*, and whose lengths follow a Poisson distribution with mean value *t* and have a maximum value of *K*. We denote by ${\mathrm{Poiss}}(t)$ a Poisson random variable with mean *t*. By the Chernoff bound^28^, we have

16 $$\begin{aligned} {\mathrm{P}}({\mathrm{Poiss}}(t) > K) < \frac{(et)^K e^{-t}}{K^K}, \end{aligned}$$

and if $K = \frac{\log \epsilon ^{-1}}{\log \log \epsilon ^{-1}}$, then for $t < K / \log \epsilon ^{-1}$,

17 $$\begin{aligned} {\mathrm{P}}({\mathrm{Poiss}}(t) > K) < \epsilon . \end{aligned}$$

As a result, we have

18 $$\begin{aligned} \rho _{t, s}(v) - \epsilon< \rho (K)_{t, s}(v) < \rho _{t, s}(v) \end{aligned}$$

with probability at least $1-\epsilon$, for each node *v* in the network. Our analysis of the approximation error of (11) relies on the Chernoff bound as summarized in Lemma 1^20^.

### **Lemma 1**

*Let* $X_i$ *be independent Bernoulli random variables with* $X = \sum _{i=1}^r X_i$. *Then*,

1. *for* $$0< \epsilon < 1$$, $${\mathrm{P}}( X< (1 - \epsilon ) r {\mathrm{E}}[X ]) < \exp \left( -\frac{\epsilon ^2}{2} r {\mathrm{E}}[X ]\right) $$,
2. *for* $$0< \epsilon < 1$$, $${\mathrm{P}}( X > (1 + \epsilon ) r {\mathrm{E}}[X ]) < \exp \left( -\frac{\epsilon ^2}{4} r {\mathrm{E}}[X ]\right) $$,
3. *for* $$c \ge 1$$, $${\mathrm{P}}( X > (1 + c) r {\mathrm{E}}[X ]) < \exp \left( -\frac{c}{2} r {\mathrm{E}}[X ]\right) $$.

We now state our bound on the approximation error of our approximate heat kernel PageRank vector.

### **Theorem 1**

*Let* *G* *be a graph and* *s* *be a node in a local cluster* $C \subset V$ *satisfying* $\Phi (C) < \phi$, ${\mathrm{vol}}(C)/{\mathrm{vol}}(G) < \psi$ *and*

19 $$\begin{aligned} \phi \psi < 2 \epsilon \cdot \left( \frac{\log \log \epsilon ^{-1}}{\log \epsilon ^{-1}} \right) ^2. \end{aligned}$$

*Let* $\rho _{t, s}$ *be the heat kernel PageRank vector with* ${\varvec{1}}_u$ *as the starting distribution. For any* $0< \epsilon < 1$, *Algorithm* 2 *outputs an approximate heat kernel PageRank vector* ${\hat{\rho }}_{t, s}$ *satisfying*

20 $$\begin{aligned} (1 - \epsilon ) \rho _{t, s}(v) - 2\epsilon< {\hat{\rho }}_{t, s}(v) < \rho _{t, s}(v), \end{aligned}$$

*for all nodes* $v \in C$, *with a probability of at least* $1 - \epsilon$. *The running time of the algorithm is* ${\mathrm{O}}\left( \frac{\log \epsilon ^{-1} \log \varsigma }{\epsilon ^3 \log \log \epsilon ^{-1}} \right)$, *where* $\varsigma$ *is the volume of the local cluster* *C*.

The proof of Theorem 1 is given in “Appendix A”. Theorem 1 states that, as long as the local cluster *C* of interest is sufficiently small compared with the whole network *G*, then Algorithm 2 provides a good approximation of $\rho _{t, s}$ for all nodes in the local cluster *C*. Many real-world networks are massive, and we are usually interested only in local structures near the seed node of interest, namely a small local cluster. The results in Theorem 1 indicate that we can efficiently compute an approximate heat kernel PageRank for a small local subgraph when analyzing a massive network, and thus probe the local structures near the seed node without incurring huge computational loads.

## Finding a good local cluster

Our local clustering algorithm finds a subset of nodes near the seed node *s* by performing a sweep on the approximate heat kernel PageRank vector ${\hat{\rho }}_{t, s}$. We now show that the ranking deduced from our approximate heat kernel PageRank can be used to find a good local cluster near the seed node. Specifically, by performing a sweep on ${\hat{\rho }}_{t, s}$ with an appropriate seed node *s* in a subgraph *C* with $\Phi (C) < \phi$, we can find a subset of nodes with a conductance value of at most ${\mathrm{O}}(\sqrt{\phi })$. The performance guarantee for the local cluster estimated by our proposed algorithm relies on the results summarized in Lemma 2^21,29^.

### **Lemma 2**

*In a graph* *G* *with a subset* *C* *with* ${\mathrm{vol}}(C) = \varsigma \le {\mathrm{vol}}(G) / 4$, *the set of* *u* *in* *C* *satisfying*

21 $$\begin{aligned} \frac{1}{2} e^{-t \Phi ^*(C)} \le \rho _{t, u}(C) \le \sqrt{\varsigma } \exp (-t \Phi _{\varsigma }(\rho _{t, u})^2 / 4) \end{aligned}$$

*has a volume of at least* $\varsigma / 4$, *provided* $t \ge 1 / (\Phi ^*(C))^2$, *where*

22 $$\begin{aligned} \Phi ^*(C) := \min _{T \subset C} \Phi (T). \end{aligned}$$

The ranking deduced from the approximate heat kernel PageRank vector ${\hat{\rho }}_{t, s}$ is not very different from that of a true vector $\rho _{t, s}$ in the local cluster *C*, as demonstrated by our bound on approximation errors in Theorem 1 (see “Computational complexity” section for an experimental analysis). For nodes in the subgraph *S* but not in the local cluster *C*, we note that we discard all simulated random walks that exit *S* and thus ${\hat{\rho }}_{t, s}(v) < \rho _{t, s}(v)$ for any $v \in S {\setminus } C$. However, unlike the local cluster *C*, the sampled subgraph *S* is not guaranteed to have few boundary edges. Thus the approximation errors in $S {\setminus } C$ are not bounded from below as in Theorem 1. We note in addition that the approximation errors decrease the values of the approximate PageRank vector ${\hat{\rho }}_{t, s}$. Thus, compared with the ranking deduced from the true PageRank vector $\rho _{t, s}$, the ranking deduced from the approximate PageRank vector ${\hat{\rho }}_{t, s}$ keeps the relative ordering of the nodes in *C* and also keeps the nodes in $S {\setminus } C$ behind the nodes in *C*. In other words, the segments $S_i$ with small *i*’s would more likely correspond to subsets of *C*. Then, as shown by Chung and Simpson^21^, we can apply the results in Lemma 2 to our approximate heat kernel PageRank vector ${\hat{\rho }}_{t, s}$. Therefore by the bounds in (20) and Lemma 2,

23 $$\begin{aligned} {\hat{\rho }}_{t, s}(C) > (1 - \epsilon ) \rho _{t, s}(C) - 2c\epsilon , \end{aligned}$$

where $c = |C |$ is the number of nodes in *C*, and in particular,

24 $$\begin{aligned} \frac{1}{2} (1 - \epsilon ) e^{-t \Phi ^*(C)} - 2c\epsilon< {\hat{\rho }}_{t, s}(C) < \sqrt{\varsigma } \exp (-t \Phi _{\varsigma }({\hat{\rho }}_{t, s})^2 / 4) \end{aligned}$$

for a set of nodes with volume at least $\varsigma / 2$. We now present our performance guarantee using the bounds in (23).

### **Theorem 2**

*Suppose that* *G* *is a network and* $C \subset G$ *is a local cluster with* ${\mathrm{vol}}(C) = \varsigma$, $|C |= c$, *and* $\Phi (C) \le \phi$. *There exists a subset* $C_t \subset C$ *with* ${\mathrm{vol}}(C_t) \ge \varsigma / 2$, *such that for any node* $s \in C_t$, *the approximate heat kernel PageRank vector* ${\hat{\rho }}_{t, s}$ *computed by Algorithm* 2 *satisfying the following two assumptions*

25 $$\begin{aligned}&t = \phi ^{-1} \left( \log (2\sqrt{\varsigma } + 8c\epsilon ) - \log (1 - \epsilon ) \right) , \end{aligned}$$

26 $$\begin{aligned}{}&\Phi _{\varsigma }({\hat{\rho }}_{t, s})^2 < \frac{4}{t} \log 2, \end{aligned}$$

*induces a ranking for which a sweep finds a set with* $\varsigma$-*local Cheeger ratio at most* $\sqrt{8\phi }$.

The proof of Theorem 2 is given in “Appendix B”. Theorem 2 implies that, we can find a good local cluster by choosing parameters *t* and $\epsilon$ following the assumptions in (25) and (26). Specifically, for a subset *C* with Cheeger ratio bounded by $\phi$, for any seed node *s* in $C_t \subset C$ as defined in Theorem 2, performing a sweep on the output of Algorithm 2 yields a local cluster with conductance at most $\sqrt{8\phi }$.

## Computational complexity

Sampling a subgraph using Algorithm 1 is sublinear in terms of the expected volume of the subgraph, which is in proportion to the expected volume $\varsigma$ of the local cluster. According to the construction of the sampling algorithm, multiple nodes can be added to the subgraph in each round. Hence the number of computations required for subgraph sampling is sublinear in terms of $\varsigma$.

We adopt the assumptions that drawing from a probability distribution with bounds and performing a random walk step both require constant time. For each of the *r* iterations, at most $K = \frac{\log \epsilon ^{-1}}{\log \log \epsilon ^{-1}}$ random walk steps are simulated, and the number of simulated random walks is $r = \frac{16}{\epsilon ^3}\log \varsigma$. Thus the number of computations for computing the approximate heat kernel PageRank is ${\mathrm{O}}\left( \frac{\log \epsilon ^{-1} \log \varsigma }{\epsilon ^3\log \log \epsilon ^{-1}} \right)$.

Performing a sweep on the approximate heat kernel PageRank vector ${\hat{\rho }}$ involves sorting the nodes according to their probability per degree and calculating the conductance of each segment. Recall that there are *m* nodes in the sampled subgraph *S*, which is in proportion to the expected size *c* of the local cluster *C*. Thus the number of computations for performing a sweep over ${\hat{\rho }}$ is ${\mathrm{O}}(m\log m) = {\mathrm{O}}(c\log c)$.

To summarize, the computational work of our local clustering algorithm is dominated by computing the approximate heat kernel PageRank, and the total number of computations is ${\mathrm{O}}\left( \frac{\log \epsilon ^{-1} \log \varsigma }{\epsilon ^3\log \log \epsilon ^{-1}} \right)$. We emphasize that, unlike some other approximation algorithms based on iterations, our proposed algorithm is based on simulated random walks, and thus allows parallel computations. As a result, in practice our proposed algorithm can be transformed into distributed computation tasks, thus improving running time^30^.

## Numerical experiments

We test our proposed local clustering algorithm on benchmark networks with ground-truth communities and compare the results to those of other existing local clustering algorithms. We will compare the clustering results in terms of the accuracy and the conductance of the estimated clusters.

### Benchmark

We use benchmark networks to compare our local clustering algorithm with the algorithm by Chung and Simpson^21^, the TEA+ algorithm^24^, and with the results of performing a sweep on the exact heat kernel PageRank with maximum length *K*

27 $$\begin{aligned} \rho (K)_{t,s} = \sum _{k\le K}e^{-t}\frac{t^k}{k!}\chi _sP^k. \end{aligned}$$

Benchmark networks are computer-generated graphs with ground-truth communities. We use the Lancichinetti–Fortunato–Radicchi (LFR) network as the benchmark for our numerical experiments^26^. In this benchmark, each computer-generated graph contains *n* nodes and is partitioned into *l* communities. The degrees of the nodes and the sizes of the ground-truth communities both follow a power-law distribution, with exponents $\gamma$ and $\beta$ respectively. The minimal and maximal degrees, $k_{\min },k_{\max }$, and the minimal and maximal community sizes, $s_{\min },s_{\max }$, are chosen to satisfy $k_{\min }<s_{\min }$ and $k_{\max }<s_{\max }$. Each node is assigned to one of the ground-truth communities, and the community structure is controlled by a mixing parameter $\mu$. For each node, a fraction $1-\mu$ of its edges connect to other members of the same community chosen at random, and the remaining fraction $\mu$ of its edges connect to nodes outside its community also chosen at random. In our numerical simulations, we choose as parameters $n=10000$, $l=30$, $k_{\min }=30$, $k_{\max }=300$, $\gamma =2$, $s_{\min }=50$, $s_{\max }=2000$, and $\beta =1.1$, and we vary the mixing parameter $\mu$ from 0.1 to 0.4. For each mixing parameter, we generate 400 graphs independently. For each generated graph, we randomly choose one of the ground-truth communities and then randomly choose one node in this community as a seed node. Then we apply the local clustering algorithms to find a local community of this seed node.

### Subgraph sampling

**Figure 1:**
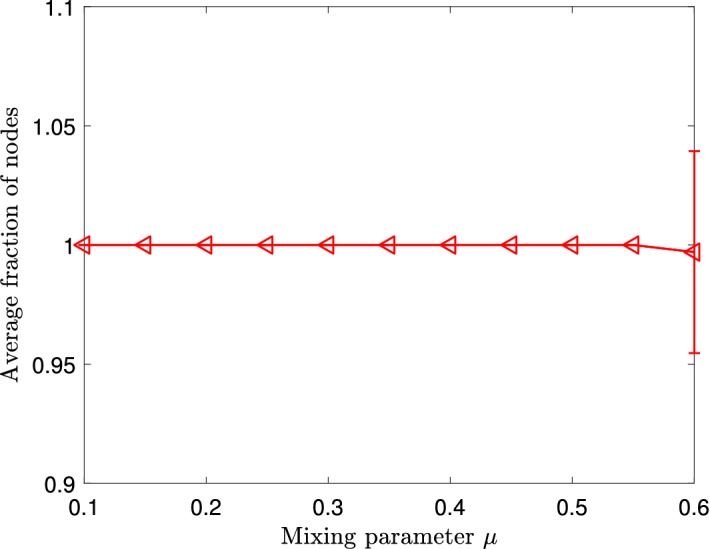
Average fraction of nodes in the chosen local cluster that is also in the sampled subgraph in the Lancichinetti–Fortunato–Radicchi benchmark. Vertical error bars represent standard deviations.

**Figure 2:**
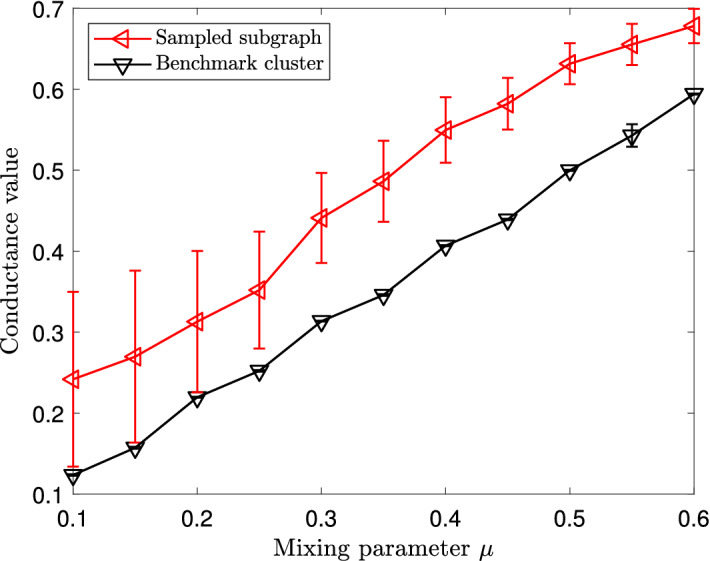
Average conductance values of sampled subgraphs in the Lancichinetti–Fortunato–Radicchi benchmark. Vertical error bars represent standard deviations.

As the first part of our numerical experiments, we evaluate the performance of our subgraph sampling algorithm. We vary the mixing parameter from 0.1 to 0.6 to better demonstrate the performance of this algorithm. As a measure of goodness, we choose the fraction of nodes in the chosen local cluster that is also in the sampled subgraph

28 $$\begin{aligned} {\text {fraction of nodes sampled}} := \frac{|C \cap S |}{|C |}, \end{aligned}$$

where *C* indicates the local cluster chosen, and *S* indicates the local subgraph found by our subgraph sampling algorithm. We choose $2{\mathrm{vol}}(C)$ as the expected size of the sampled subgraph, and note that in practice we can choose a larger expected size when working with large networks.

The average fraction with varying mixing parameters of our subgraph sampling algorithm is shown in Fig. 1. The sampled subgraph contains all the nodes of the chosen local cluster as the value of the mixing parameter $\mu$ varies from 0.1 to 0.55, and starts to degrade slightly only after the value of $\mu$ exceeds 0.55. Thus, our subgraph sampling algorithm always finds a local subgraph that contains the local cluster of interest as a subgraph, as long as the nodes in this local cluster are more strongly connected internally than to nodes outside this cluster.

We also evaluate the goodness of the local subgraphs found by our subgraph sampling algorithm by measuring the conductance values of these local subgraphs. Figure 2 shows the average conductance values of sampled subgraphs with varying mixing parameters $\mu$. When $\mu = 0.1$, the average conductance value of sampled subgraphs is around 0.2. The average conductance value gradually increases as $\mu$ becomes larger, reaching around 0.65 when $\mu =0.6$. We note that, for each local cluster, the mixing parameter is the average fraction of edges that connect to nodes in other clusters, and so approximately equals the conductance values of these local clusters. We also show in Fig. 2 the average conductance value of benchmark clusters. The average conductance value of the sampled subgraphs grows almost in parallel with that of the benchmark clusters, with an increase of about 0.1. Thus our subgraph sampling algorithm finds local subgraphs with conductance values slightly greater than those of the local clusters of interest.

### Local clustering

**Figure 3:**
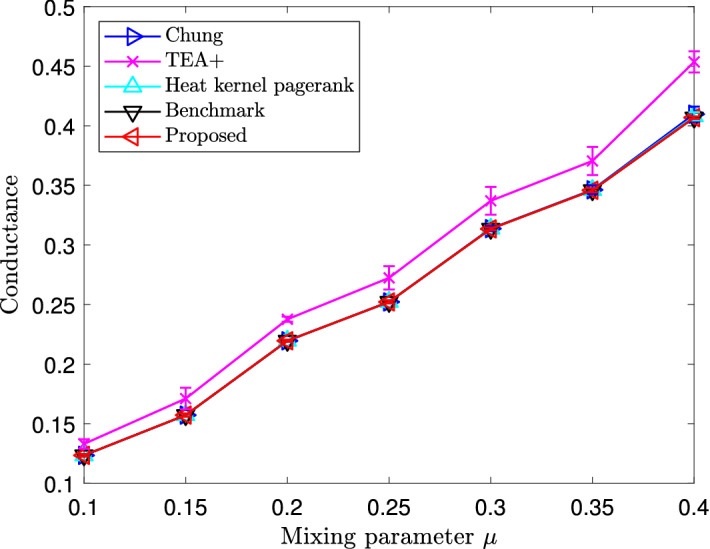
Average conductance values of local clusters estimated by performing sweeps on different heat kernel PageRank vectors in the Lancichinetti–Fortunato–Radicchi benchmark. Vertical error bars represent standard deviations.

**Figure 4:**
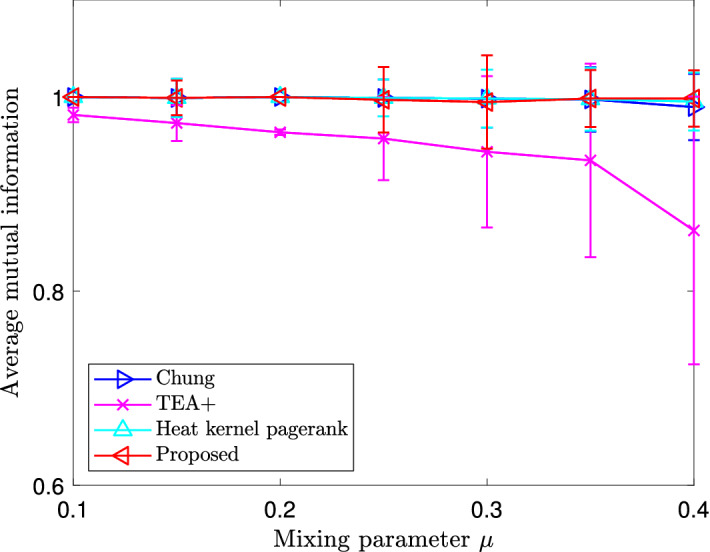
Average mutual information of local clusters estimated by different local clustering methods with benchmark communities in the Lancichinetti–Fortunato–Radicchi benchmark. Vertical error bars represent standard deviations.

As a second step, we compare the goodness of local clusters found by these local clustering algorithms. We first compute the conductance values of estimates by performing sweeps on different PageRank vectors. The average conductance values of local-cluster estimates with various mixing parameters are shown in Fig. 3, where the term “heat kernel” refers to performing sweeps on the heat kernel PageRank vector with maximum length, as in (13), and “benchmark” refers to the conductance values of the chosen clusters in the benchmark networks, averaged over experiments with the same mixing parameters. As we can see, for all values of the mixing parameter $\mu$, all methods except the TEA+ algorithm yield conductance values equal to those of benchmark clusters. In particular, our proposed algorithm, despite the approximation errors in computing the heat kernel PageRank, finds local clusters as good as the ground-truth clusters, in terms of conductance. The clusters estimated by the TEA+ algorithm all have conductance values greater than those of the benchmark clusters.

We evaluate the similarity between two clusters $C_1$ and $C_2$ using the normalized mutual information^31,32^

29 $$\begin{aligned} I_n({\mathcal {C}}_1,{\mathcal {C}}_2) := \frac{\sum _{c_1\in {\mathcal {C}}_1,c_2\in {\mathcal {C}}_2}p(c_1,c_2)\log \frac{p(c_1,c_2)}{p(c_1)p(c_2)}}{\frac{1}{2}{\mathcal {H}}({\mathcal {C}}_1) +\frac{1}{2}{\mathcal {H}}({\mathcal {C}}_2)}, \end{aligned}$$

where ${\mathcal {C}}_1 = (C_1, {\overline{C}}_1)$ and ${\mathcal {C}}_2 = (C_2, {\overline{C}}_2)$ are the bipartitions of the network induced by the clusters $C_1$ and $C_2$, respectively, *p*(*c*) is the probability that a randomly chosen node belongs to a subgraph *c*, $p(c_1, c_2)$ is the probability that a randomly chosen node belongs to both subgraphs $c_1$ and $c_2$, and ${\mathcal {H}}({\mathcal {C}})$ is the Shannon entropy, defined as

30 $$\begin{aligned} {\mathcal {H}}({\mathcal {C}}) := -\sum _{c\in {\mathcal {C}}}p(c)\log p(c). \end{aligned}$$

By the above definition, the mutual information $I_n({\mathcal {C}}_1,{\mathcal {C}}_2)$ equals 1 if the two subgraphs $C_1$ and $C_1$ are identical, and equals 0 if they are independent. In our numerical experiments, $C_1$ is the ground-truth community specified by the benchmark and chosen in our experimental setup, and $C_2$ is the local cluster detected by different local clustering algorithms.

The average mutual information values with varying mixing parameters of different local clustering algorithms are shown in Fig. 4. The proposed local clustering algorithm maintains almost complete mutual information in all experiments, demonstrating that the approximation errors in computing the heat kernel PageRank do not degrade performance in estimating local clusters. In other words, our proposed approximate heat kernel PageRank vector is accurate enough for finding good clusters. For other methods, when $\mu$ is small, all methods yield a good estimate of the ground-truth local community, achieving almost complete mutual information and so detecting correct clusters. When $\mu$ increases, the average mutual information of the output of the TEA+ algorithm clearly decreases, demonstrating that the clusters estimated by this algorithm deviate from the ground-truth cluster.

To conclude, even though our proposed approximate heat kernel PageRank might suffer from increased approximation errors due to discarding part of the simulated random walks, the performance of our local clustering algorithm is not significantly influenced by these errors. In fact, our local clustering algorithm achieves state-of-the-art performance on benchmark networks; it always finds local clusters that are as good as benchmark clusters and clusters estimated by other methods. Our proposed approximate computation of the heat kernel PageRank is accurate enough to find good local clusters.

## Application to real-world networks

### College football network

**Figure 5:**
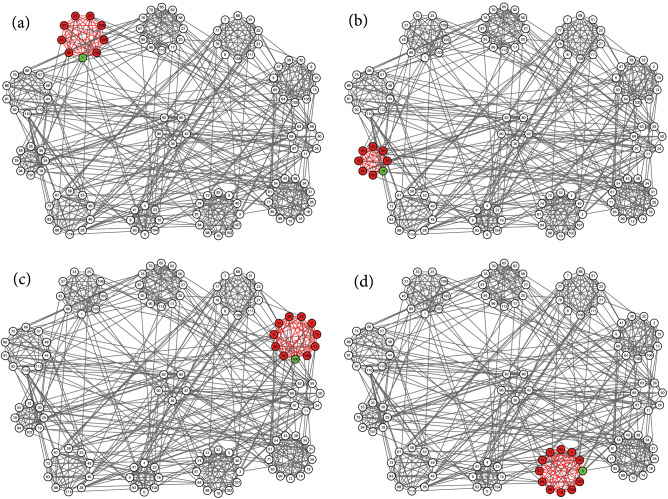
Identified local communities (red) of college football teams. Circles of nodes indicate the conferences of football teams. The five nodes placed in the middle represent five independent teams. Each of the subfigures shows the local clustering result generated by the proposed method, using seed nodes (green) 1, 19, 100, and 3.

**Figure 6:**
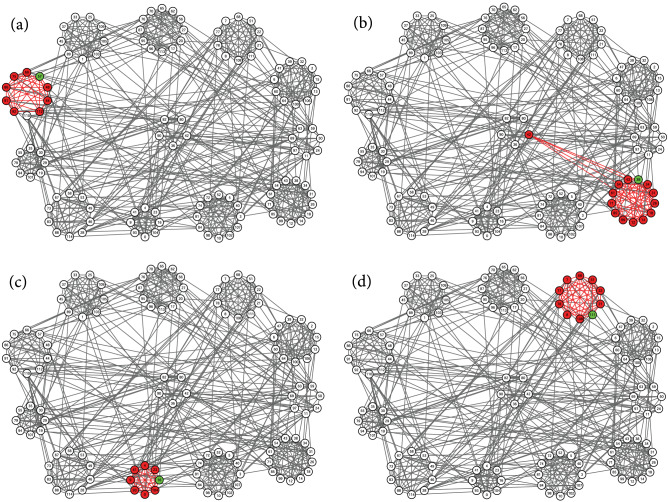
Identified local communities (red) of college football teams. Circles of nodes indicate the conferences of football teams. The five nodes placed in the middle represent five independent teams. Each of the subfigures shows the local clustering result generated by the proposed method, using seed nodes (green) 57, 38, 16, and 111.

**Figure 7:**
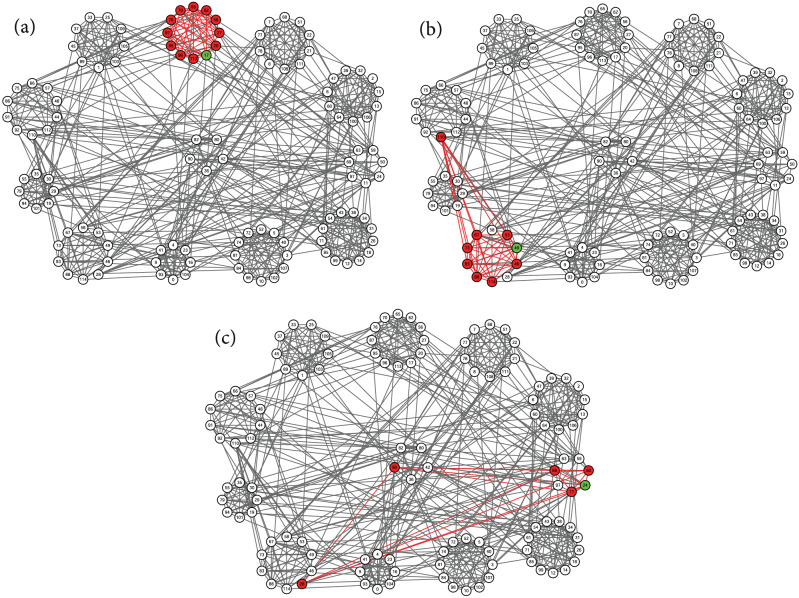
Identified local communities (red) of college football teams. Circles of nodes indicate the conferences of football teams. The five nodes placed in the middle represent five independent teams. Each of the subfigures shows the local clustering result generated by the proposed method, using seed nodes (green) 17, 49, and 24.

We first apply the proposed local clustering algorithm to a real-world network with a known labeled community structure. This network represents the schedule of American football games between 116 football teams from Division IA colleges during the regular season in Fall 2000^25^. A visualization of this network is shown in Figs. 5, 6, and 7 using Cytoscape^33^, where nodes represent teams and edges mean that there exist regular season games between the pair of teams connected. These football teams belong to 12 conferences, which we use as reference ground-truth communities. Every conference contains around 8 to 12 teams, represented as circles of nodes in our visualization. The five teams in the middle of the figure are not involved in any of the circles. These independent teams do not belong to any conference. In principle, teams tend to play more games within conferences than between conferences, which generates more edges within each conference.

Each subfigure of Figs. 5, 6, and 7 shows the local clusters found by the proposed method, using different seed nodes. We choose one node from each conference as a seed node, and use the size of the conference as the expected size of a local cluster. We mark the seed node in green, and mark nodes found to be a member of the local cluster in red. We also highlight in red the edges between the members of the found local cluster. In most cases, as the local cluster, the proposed algorithm correctly finds the conference to which the seed node belongs.

We also observe in some cases that the resulting local cluster does not fit very well with the reference communities labeled by conferences. In Fig. 6a, node 110 is not included in the local cluster of the seed node 57, which is reasonable if we carefully inspect the network connections of this node. There are no connections between node 110 and any other members of this conference; instead, this node forms several connections with almost all members of the conference at eight o’clock. Thus it is no wonder that node 110 is identified as part of the local cluster of seed node 49, as shown in Fig. 7b. We also note that the independent team node 42 is regarded as part of the local cluster of seed node 38, as shown in Fig. 6b. This team plays regular games with four teams from the conference at four o’clock, and with, at most, one team each from any other conference. So our algorithm identifies it as part of the detected local cluster. Moreover, as seen in Fig. 7b, two teams, nodes 28 and 58 of the conference at eight o’clock, are not included in the local cluster of seed node 49. But node 28 has no connections within this conference, while at the same time it maintains several connections outside this conference, especially to the four teams colored in the conference at three o’clock, as shown in Fig. 7c. This pattern of connections explains why this team is included in the local cluster of seed node 24. On the other hand, the team of node 58 plays regular games with only two teams within the conference at eight o’clock, but plays regular games with one or two teams from four other conferences and independent teams. Due to such an atypical connection pattern among multiple conferences, this node is not included in any local cluster in our experiment. Similar phenomena are also observed for the uncolored nodes in the conference at three o’clock, as shown in Fig. 7c, and thus these nodes are also left outside any local clusters. By contrast, the independent team of node 90 plays regular games with all the colored teams in this conference, and so is included in this local cluster.

In conclusion, our proposed local clustering algorithm perfectly reflects the local community structure around the selected seed nodes, established by regular-season-game association. In addition, our algorithm detects the lack of associations within conferences, as reflected by network connections.

### YouTube social network

**Figure 8:**
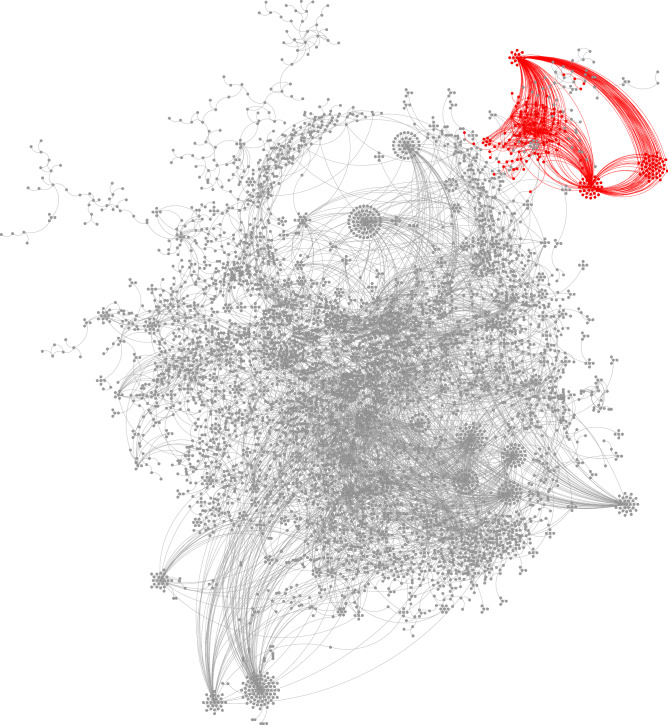
Local community (red) detected in the YouTube social network using the proposed method. Here we show only 5846 nodes among the total 1,134,890 nodes (about $0.5\%$ fraction) that are most close to the seed node.

Our second example is a social network on the video-sharing website YouTube^34^. In the YouTube social network, nodes represent users and edges represent friendships formed by users with each other. The whole network is composed of 1,134,890 nodes and 2,987,624 edges. In this network, users can create groups which other users can join, and these user-defined groups are considered as reference “ground-truth” communities^34^. We apply the proposed clustering method to one of these user-defined groups composed of 121 users, which has a conductance value of 0.2030. We randomly choose one of the group members as the seed node, and then find a local cluster of this seed node. The estimated local cluster is composed of 225 nodes, contains all 121 users in the user-defined group, and has a conductance value of 0.0168.

The local cluster found by our algorithm is visualized in Fig. 8 using Cytoscape^33^, with members of the estimated local cluster in red. Due to limited graphical processing capabilities, we show only 5,846 nodes (about $0.5\%$ of the total number of nodes) and the edges between these nodes that are closest to the seed node. This visualized subgraph is chosen using the subgraph sampling algorithm in Algorithm 1. The YouTube social network is relatively sparse and contains several locally dense groups, shown as small circles of nodes. The estimated local cluster contains three such dense groups, visualized as the three circles of nodes at top right. Two of these groups have obvious hubs, and other members surrounding them and connected only to them by edges. The other group, on the right, does not show such concentration and forms edges among its members uniformly. These three groups have multiple edges between them, and most of these edges are linked to the hubs. Some scattered nodes have many connections with these three groups, especially with the hubs of these groups, and thus these nodes are also identified as part of the local cluster.

### DBLP collaboration network

**Figure 9:**
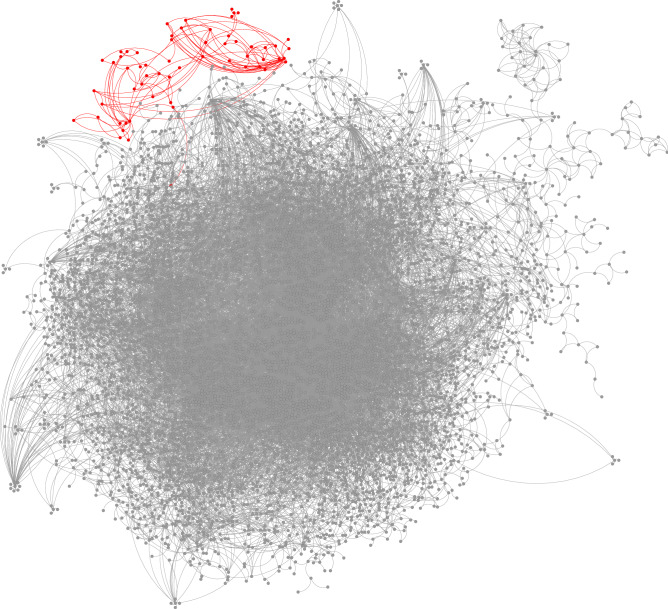
Local community (red) detected in the DBLP collaboration network using the proposed method. Here we show only 6342 nodes among the total 317,080 nodes (about $2\%$ fraction) that are closest to the seed node.

Our third example is a collaboration network on the DBLP computer science bibliography, which provides a comprehensive list of research papers in computer science^34^. In the DBLP collaboration network, nodes represent authors of these research papers, and two authors are connected by an edge if they collaborate and publish at least one research paper together. The whole network is composed of 317,080 nodes and 1,049,866 edges. The reference “ground-truth” communities are defined by publication venues, for example, journals or conferences, and authors who have published research papers in a certain journal or conference are considered to form a reference community^34^. The publication-venue group we choose is composed of 36 nodes, and has a conductance value of 0.1034. As the seed node, we randomly choose one of the group members, and then apply our proposed local clustering algorithm to find a local cluster of this seed node. The estimated local cluster is composed of 59 nodes and contains all 36 authors in the publication-venue group, and it has a conductance value of 0.0308.

The local cluster found by our algorithm is visualized in Fig. 9 using Cytoscape^33^, with members of the estimated local cluster colored in red. As before, here we show only 6,342 nodes (about $2\%$ of the total number of nodes) and the edges between these nodes that are most close to the seed node chosen using our proposed subgraph sampling algorithm. As shown in the figure, the DBLP collaboration network has a much denser connection pattern than the YouTube social network, and does not show explicit local groups. But our local clustering algorithm successfully finds a small research community at the top of the visualization, with dense collaborations among group members, and only four external links, all made by one group member. Thus our algorithm finds a research group in which all the members but one collaborate only with other group members and not with researchers outside the group.

### Amazon product co-purchasing network

**Figure 10:**
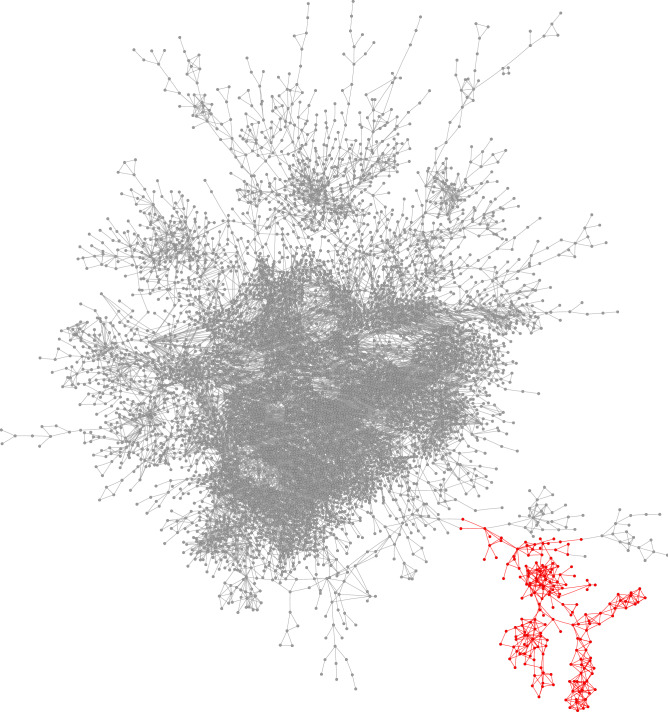
Local community (red) detected in the Amazon product co-purchasing network, using the proposed method. Here we show only 6701 nodes among the total 334,863 nodes (about $2\%$ fraction) that are closest to the seed node.

Our fourth example is a product co-purchasing network of Amazon users. This network was collected by crawling the Amazon website and is based on a co-purchasing feature, “Customers Who Bought This Item Also Bought,” of the Amazon website^34^. In the Amazon product co-purchasing network, nodes represent products, and two products are connected by an edge if these two products are frequently co-purchased. The whole network is composed of 334,863 nodes and 925,872 edges. The reference “ground-truth” communities are defined by the product categories provided by Amazon. The product-category group we choose is composed of 96 products, and has a conductance value of 0.0235. The seed node is chosen as a random product in this category, and we then find a local cluster of this seed node using our proposed local clustering algorithm. The estimated local cluster is composed of 184 nodes, contains all 96 products in the product-category group, and has a conductance value of 0.0064.

The local cluster found by our algorithm is visualized in Fig. 10 using Cytoscape^33^, with members of the estimated local cluster colored in red. As before, we show only 6,701 nodes (about $2\%$ of the total number of nodes) and the edges between these nodes that are closest to the chosen seed node using our proposed subgraph sampling algorithm. As can be seen, the Amazon co-purchasing network has a giant and dense group of connections in the middle, with many branches that are relatively sparse and contain some locally dense groups. Our algorithm finds a local cluster that is part of one of these branches. The estimated local cluster contains approximately three local dense groups, with multiple edges connecting them. The estimated local cluster has only two edges connecting itself to the main part of the network, effectively separating it from the giant dense group in the middle. We also note two other dense groups on this branch, but each has only one edge connected to our estimated local cluster. As a result, these two dense groups are not regarded as part of our estimated local cluster.

## Conclusion and discussion

In this paper, we introduced a local algorithm for computing approximate heat kernel PageRank vectors, and based on this algorithm we devised a novel local clustering algorithm. We restricted our computation of heat kernel PageRank vectors to within a local subgraph near the seed node, discarding simulated random walks that depart from this subgraph. As a result, the number of computations required to achieve a good approximation is no longer dependent on the size of the whole network, and is determined only by the expected size of the local cluster of interest. We showed that, under reasonable assumptions, the approximation error caused by restricting simulated random walks to within a local subgraph is bounded by a probabilistic guarantee. We also presented a greedy local search algorithm for finding a local subgraph near the seed node. The numbers of computations required for subgraph sampling and computing approximate heat kernel PageRank vectors are both sublinear in terms of the expected size of the local cluster of interest, and thus are both independent of the size of the whole network. Using numerical experiments, we demonstrated that the resulting local subgraph almost certainly contains the local cluster of interest as a subgraph, as long as the nodes in this local cluster are more strongly connected internally than to nodes outside this cluster. This local cluster has a conductance value only slightly greater than that of the local cluster of interest. Putting these results together, we then argued that, by performing a sweep on the proposed approximate heat kernel PageRank vector, our local clustering algorithm can find a local cluster having a small conductance value with high probability. Then we tested our local clustering algorithm on benchmark networks with well-established ground-truth communities, and on a football network with reference communities. In both cases, our local clustering algorithm achieved state-of-the-art performance, and the local clusters it found aligned well with ground-truth communities. Finally, we applied our local clustering algorithm to massive real-world networks with reference communities labeled using side information. Specifically, our algorithm provided insight into the online groupings of a video-sharing network, the collaboration patterns of a group of researchers, and the co-purchasing habits of Amazon users. In addition, our proposed approximate computation of the heat kernel PageRank is based on Monte Carlo simulation of random walks, and thus can be transformed into distributed algorithms in practical settings.

## Appendix

### A Proof of Theorem 1

#### *Proof*

The difference between our approximation in (12) and simulated random walks in (14) lies in those random walks that leave subgraph *S* first and then come back to the cluster *C*. We will instead bound the contribution from the random walks that leave *C* first and then come back. For a node *u* outside of the cluster *C*, say in another community $C'$, suppose that a random walk starting from seed node *s* reaches this node *u*. By the construction of our network model, a fraction $\mu _u$ of its edges connect to other nodes outside of its community $C'$, and thus the probability of this random walk leaving $C'$ at this step is $\mu _u$. There are $\sum _{v\notin C'}\mu _vd_v$ edges that this random walk can choose if it leaves $C'$, and among them $|\partial (C) |$ edges lead to our community of interest *C*. Denote by $\nu _u$ the probability that this random walk enters *C* from node *u*, and we have

31 $$\begin{aligned} \nu _u = \mu _u \frac{|\partial (C) |}{\sum _{v\notin C'}\mu _vd_v} \approx \mu _u \frac{|\partial (C) |}{\sum _v\mu _vd_v}, \end{aligned}$$

where the approximation becomes exact when the network is large enough. Then the average probability that a random walk enters *C*, denoted as $\nu$, is

32 $$\begin{aligned} \nu \approx \mu \frac{|\partial (C) |}{\mu {\mathrm{vol}}(V)} = \frac{|\partial (C) |}{{\mathrm{vol}}(V)} = \frac{|\partial (C) |}{{\mathrm{vol}}(C)} \cdot \frac{{\mathrm{vol}}(C)}{{\mathrm{vol}}(V)} = \phi \psi . \end{aligned}$$

The combined random variable corresponding to random walks with length at most *K* that leaves the cluster *C* exactly once and then comes back exactly once is given as

33 $$\begin{aligned} Z^v_1= \,& {} \sum _{k\le K}e^{-t}\frac{t^k}{k!}X_k^v\sum _{j=1}^{k-2} (1-\mu _{t_1})\cdots (1-\mu _{t_j}) (1-\nu _{s_1})\cdots (1-\nu _{s_{k-2-j}})\mu _{t_{j+1}}\nu _{s_{k-1-j}}, \end{aligned}$$

where $t_1,\dots ,t_j,t_{j+1}$ are nodes in *C* that are visited in a *k*-step random walk and $s_1,\dots ,s_{k-2-j},s_{k-1-j}$ are nodes outside *C* that are visited in a *k*-step random walk. Then the expected contribution to $\rho (K)_{t,s}(v)$ from random walks that leave *C* once and then enter *C* once is

34 $$\begin{aligned} {\mathrm{E}}[Z^v_1 ]= \sum _{k\le K}e^{-t}\frac{t^k}{k!}{\mathrm{E}}[X_k^v ]\sum _{j=1}^{k-2}(1-\mu )^j(1-\nu )^{k-2-j}\mu \nu . \end{aligned}$$

Now consider the more general case that random walks leave the cluster *C* for exactly $\ell$ times, enter the cluster *C* for exactly $\ell$ times, and end at node *v*, where $\ell$ is a positive integer less than *k*/2. The random variable corresponding to these random walks is thus

35 $$\begin{aligned} Z^v_{\ell }= \,& {} \sum _{k\le K}e^{-t}\frac{t^k}{k!}X_k^v \sum _{j=1}^{k-2\ell } \prod _{r=1}^j(1-\mu _{t_r})\prod _{q=1}^{k-2\ell -j}(1-\nu _{s_q}) \prod _{w=1}^{\ell }\mu _{t_{j+w}}\nu _{s_{k-2\ell -j+w}}. \end{aligned}$$

Similarly, the expected contribution to $\rho (K)_{t,s}(v)$ from these random walks can be written as

36 $$\begin{aligned} {\mathrm{E}}[Z^v_{\ell } ]= \,& {} \sum _{k\le K}e^{-t}\frac{t^k}{k!}{\mathrm{E}}[X_k^v ]\sum _{j=1}^{k-2\ell }(1-\mu )^j(1-\nu )^{k-2\ell -j}\mu ^{\ell }\nu ^{\ell }. \end{aligned}$$

Define $Z^v$ as $\sum _{\ell } Z_{\ell }^v$, and then the expected contribution to $\rho (K)_{t, s}(v)$ of all random walks that first leave the cluster *C* and then come back and end at node *v* is

37 $$\begin{aligned} {\mathrm{E}}[Z^v ]= \sum _{\ell =1}^{K/2}{\mathrm{E}}[Z^v_{\ell } ]= \,& {} \sum _{\ell =1}^{K/2}\sum _{k\le K}e^{-t}\frac{t^k}{k!}{\mathrm{E}}[X_k^v ]\sum _{j=1}^{k-2\ell }(1-\mu )^j(1-\nu )^{k-2\ell -j}\mu ^{\ell }\nu ^{\ell }. \end{aligned}$$

Noting that $1-\mu$, $1-\nu$, $\mu$ are all less than 1, we have

38 $$\begin{aligned} {\mathrm{E}}[Z^v ]\le \,& {} \sum _{k\le K} e^{-t}\frac{t^k}{k!}{\mathrm{E}}[X_k^v ]\sum _{\ell =1}^{K/2} (K-2\ell ) \nu ^{\ell } \le \rho (K)_{t, s}(v) \cdot \frac{K^2 \nu }{8} \le \rho (K)_{t, s}(v) \cdot \frac{\epsilon }{2}, \end{aligned}$$

where the last inequality comes from $K^2\nu / 2 < \epsilon$ and $K = \frac{\log \epsilon ^{-1}}{\log \log \epsilon ^{-1}}$. Suppose that we perform $r > \frac{16}{\epsilon ^3}\log \varsigma$ simulated random walks, where $\varsigma$ is the volume of the sampled subgraph *S*. For every component with $\rho (K)_{t, s}(v) > \epsilon$, by the third part of Lemma 1, we have

39 $$\begin{aligned} {\mathrm{P}}(Z^v> r \epsilon \rho (K)_{t, s}(v))\le \,& {} {\mathrm{P}}\left( Z^v> r \left( \frac{\epsilon }{2} \rho (K)_{t, s}(v) + {\mathrm{E}}[Z^v ]\right) \right)  \\= \,& {} {\mathrm{P}}\left( Z^v > r \left( 1 + \frac{\epsilon \rho (K)_{t, s}(v)}{2{\mathrm{E}}[Z^v ]} \right) {\mathrm{E}}[Z^v ]\right)  \\ \le \,& {} \exp \left( -\frac{\epsilon \rho (K)_{t, s}(v)}{4{\mathrm{E}}[Z^v ]} r {\mathrm{E}}[Z^v ]\right)  \\ \le \,& {} \exp \left( -\frac{\epsilon }{4} r \rho (K)_{t, s}(v) \right)  \\< \,& {} \exp \left( -\frac{r\epsilon ^2}{4} \right)  \\< \,& {} \varsigma ^{-4/\epsilon }. \end{aligned}$$

For every component with $\rho (K)_{t, s}(v) < \epsilon$, by the third part of Lemma 1, we have

40 $$\begin{aligned} {\mathrm{P}}(Z^v> r \epsilon )\le \,& {} {\mathrm{P}}\left( Z^v> r\left( \frac{\epsilon }{2} + {\mathrm{E}}[Z^v ]\right) \right)  \\= \,& {} {\mathrm{P}}\left( Z^v > \left( 1 + \frac{\epsilon }{2{\mathrm{E}}[Z^v ]} \right) r {\mathrm{E}}[Z^v ]\right)  \\< \,& {} \exp \left( -\frac{\epsilon }{4{\mathrm{E}}[Z^v ]} r {\mathrm{E}}[Z^v ]\right)  \\= \,& {} \exp \left( -\frac{r\epsilon }{4} \right)  \\< \,& {} \varsigma ^{-4/\epsilon ^2}. \end{aligned}$$

We note that our approximate PageRank vector equals ${\hat{\rho }}_{t, s}(v) = \rho (K)_{t, s}(v) - \frac{1}{r} Z^v$. Thus for any node *v* in the local cluster *C* with $\rho (K)_{t, s}(v) > \epsilon$, we have

41 $$\begin{aligned} (1 - \epsilon ) \rho (K)_{t, s}(v)< {\hat{\rho }}_{t, s}(v) < \rho (K)_{t, s}(v), \end{aligned}$$

with probability at least $1 - \varsigma ^{-4/\epsilon }$, and for any node *v* in the local cluster *C* with $\rho (K)_{t, s}(v) \le \epsilon$, we have

42 $$\begin{aligned} \rho (K)_{t, s}(v) - \epsilon< {\hat{\rho }}_{t, s}(v) < \rho (K)_{t, s}(v), \end{aligned}$$

with probability at least $1 - \varsigma ^{-4/\epsilon ^2}$. Thus, combined with (18), for each node *v* in the local cluster *C*,

43 $$\begin{aligned} (1 - \epsilon ) \rho _{t, s}(v) - 2\epsilon< {\hat{\rho }}_{t, s}(v) < \rho _{t, s}(v), \end{aligned}$$

with probability at least $1 - \max (\epsilon , \varsigma ^{-4/\epsilon })$, which completes our proof. $\square$

### B Proof of Theorem 2

#### *Proof*

Let $C_t$ be the set of nodes satisfying the inequality in (24). Now rearranging this inequality, we get

44 $$\begin{aligned} \exp ( -t \Phi ^*(C) ) < \frac{2}{1 - \epsilon } \left( \sqrt{\varsigma } \left( \exp \left( -t \frac{\Phi _{\varsigma }( {\hat{\rho }}_{t, s})^2}{4} \right) + 2c\epsilon \right) \right) , \end{aligned}$$

and further rearrangement of the right-hand side yields

45 $$\begin{aligned} \exp ( -t \Phi ^*(C) ) < \exp \left( -t \frac{\Phi _{\varsigma }( {\hat{\rho }}_{t, s})^2}{4} \right) \left( \frac{2\sqrt{\varsigma }}{1-\epsilon } + \frac{4c\epsilon }{1-\epsilon } \exp \left( t \frac{\Phi _{\varsigma }( {\hat{\rho }}_{t, s})^2}{4} \right) \right) . \end{aligned}$$

Then by the assumption in (26), we have

46 $$\begin{aligned} \exp ( -t \Phi ^*(C) ) < \exp \left( -t \frac{\Phi _{\varsigma }( {\hat{\rho }}_{t, s})^2}{4} \right) \left( \frac{2\sqrt{\varsigma }}{1-\epsilon } + \frac{8c\epsilon }{1-\epsilon } \right) , \end{aligned}$$

and thus

47 $$\begin{aligned} \Phi ^*(C) > \frac{\Phi _{\varsigma }({\hat{\rho }}_{t, s})^2}{4} - \frac{\log (2\sqrt{\varsigma } + 8c\epsilon ) - \log (1 - \epsilon )}{t}. \end{aligned}$$

Then by the assumption in (25), we have

48 $$\begin{aligned} \Phi ^*(C) > \frac{\Phi _{\varsigma }({\hat{\rho }}_{t, s})^2}{4} - \phi . \end{aligned}$$

Applying that fact that $\Phi ^*(C) < \phi$ and rearranging (48) yields

49 $$\begin{aligned} \Phi _{\varsigma }({\hat{\rho }}_{t, s})^2< 4\Phi ^*(C) + 4\phi < 8\phi . \end{aligned}$$

Thus a sweep over the approximate PageRank vector ${\hat{\rho }}_{t, s}$ finds a subset with Cheeger ratio at most ${\mathrm{O}}(\sqrt{\phi })$ as long as *s* is in $C_t$. $\square$
